# Association of Serum Vitamin D Levels and Ventricular Dysfunction Among Patients With Acute ST-Segment Elevation Myocardial Infarction in a Tertiary Care Hospital

**DOI:** 10.7759/cureus.109330

**Published:** 2026-05-21

**Authors:** Kartikeya Arora, Sheryl Elsa Abraham, Rahul Chopra, Manish P Parmar

**Affiliations:** 1 Internal Medicine, Subharti Medical College, Meerut, IND; 2 General Medicine, Mersey and West Lancashire Teaching Hospitals NHS Trust, Prescot, GBR; 3 Community Medicine, NAMO (National Academic Medical Organisation) Medical Education and Research Institute, Silvassa, IND; 4 General Medicine, Dr. Narendra Dharmsinh Desai Faculty of Medical Science and Research, Dharmsinh Desai University, Nadiad, IND

**Keywords:** acute coronary syndrome, ejection fraction, inflammation, left ventricular dysfunction, st-segment elevation myocardial infarction, vitamin d deficiency

## Abstract

Background

Vitamin D deficiency is common in acute coronary syndromes and has been implicated in inflammation, myocardial injury, and adverse ventricular remodeling. However, evidence linking serum 25-hydroxyvitamin D (25(OH)D) levels with early ventricular dysfunction in acute ST-segment elevation myocardial infarction (STEMI) remains limited. This study evaluated the association between serum vitamin D status and left ventricular (LV) systolic dysfunction during the index hospitalization for STEMI.

Methods

This hospital-based analytical cross-sectional study included 150 consecutive adults with acute STEMI. Serum 25(OH)D was measured within 24 hours of admission, and patients were categorized as deficient (<20 ng/mL), insufficient (20-29 ng/mL), or normal (≥30 ng/mL). Transthoracic echocardiography was performed within 48-72 hours, and LV systolic dysfunction was defined as LV* *ejection fraction (LVEF) <50%. Group comparisons were performed using ANOVA and chi-square tests. Pearson correlation assessed associations between 25(OH)D and echocardiographic/inflammatory indices. Multivariable logistic regression identified independent variables associated with LV systolic dysfunction.

Results

Vitamin D status distribution was: insufficient 46.7% (n=70), normal 33.3% (n=50), and deficient 20.0% (n=30). Mean LVEF differed significantly across groups (normal 54.0 ± 6.0%, insufficient 51.6 ± 6.8%, deficient 44.8 ± 7.9%; p<0.001). LV systolic dysfunction occurred in 12/50 (24.0%) normal, 24/70 (34.3%) insufficient, and 30/30 (100.0%) deficient patients (p<0.001). Serum 25(OH)D correlated positively with LVEF (r=0.62; p<0.001) and inversely with Wall Motion Score Index (WMSI) (r=−0.58; p<0.001) and high-sensitivity* *C-reactive protein (hs-CRP) (r=−0.52; p<0.001). On multivariable analysis, vitamin D deficiency was independently associated with LV dysfunction (adjusted OR (aOR) 6.20; 95%CI 2.35-16.36; p<0.001), along with diabetes mellitus (aOR 2.85; 95%CI 1.36-5.94; p=0.005), anterior wall MI (aOR 4.10; 95%CI 1.88-8.93; p<0.001), and hs-CRP >10 mg/L (aOR 3.05; 95%CI 1.45-6.42; p=0.003).

Conclusion

Vitamin D deficiency was strongly associated with impaired early ventricular function and higher inflammatory burden in acute STEMI and showed an independent association with LV systolic dysfunction. Measurement of serum 25(OH)D may aid early risk stratification in STEMI.

## Introduction

Acute ST-segment elevation myocardial infarction (STEMI) is a coronary artery disease (CAD) manifestation and a cause of cardiovascular disease [1]. Although there is timely reperfusion and improvement in the pharmacological management, a large percentage of patients experience left ventricular (LV) systolic dysfunction, which is a main cause for poor short- and long-term outcomes such as heart failure, arrhythmias, and premature mortality [2]. Identifying potentially changeable biological factors related to early ventricular dysfunction following STEMI is thus clinically significant [3].

Furthermore, vitamin D plays an important role in bone and mineral metabolism and has been the focus of growing interest as a controller of cardiovascular structure and function [4]. Vitamin D receptors are found on endothelial cells, cardiomyocytes, and vascular smooth muscle cells, and experimental evidence indicates that it affects myocardial contractility, calcium homeostasis, inflammatory signal transduction, oxidative stress, and neurohormonal stimulation. Specifically, it has been demonstrated that vitamin D suppresses renin-angiotensin-aldosterone activity and regulates pro-inflammatory cytokine expression pathways in myocardial injury and post-infarction remodeling [5].

Previous studies reported increased hypovitaminosis D prevalence among patients with acute coronary syndromes [6-8]. Reduced 25-hydroxyvitamin D (25(OH)D) levels were related to elevated inflammatory load, severe infarcts, endothelial dysfunction, and increased incidences of unfavorable cardiovascular outcomes [9]. Nevertheless, studies that associate the status of vitamin D with ventricular systolic dysfunction during the acute stage of the STEMI are scarce and contradictory [10,11]. Most previous studies have been conducted involving a heterogeneous population that incorporates non-STEMI (NSTEMI) or unstable angina. LV systolic dysfunction following STEMI is a result of a complex combination of irreversible myocyte necrosis, myocardial stunning, inflammation, and early adverse remodeling. Vitamin D deficiency can enhance these activities, thereby making them contribute to worse systolic performance [12]. Elucidation of this relationship can enhance risk stratification at an early stage and provide a freely measurable biomarker that can be used to theorize treatment.

The current study was conducted to evaluate the relationship between serum vitamin D level and LV systolic dysfunction in patients who presented with acute STEMI to a tertiary care hospital in Gujarat, India. In this context, we investigated the association between serum 25(OH)D status and early ventricular dysfunction among adults admitted with acute STEMI. The primary objective was to evaluate whether serum 25(OH)D categories, deficient (<20 ng/mL), insufficient (20-29 ng/mL), and normal (≥30 ng/mL), are associated with LV systolic dysfunction defined as LV ejection fraction (LVEF) <50% on echocardiography during the index hospitalization. Secondary objectives were to compare echocardiographic indices of ventricular dysfunction (including LVEF, Wall Motion Score Index (WMSI), and tricuspid annular plane systolic excursion (TAPSE)) across vitamin D categories and to assess correlations between serum 25(OH)D levels and markers of ventricular function and inflammation (high-sensitivity C-reactive protein (hs-CRP)).

## Materials and methods

Study design and setting

A hospital-based analytical cross-sectional study was conducted in the Department of General Medicine at Dr. Narendra Dharmsinh Desai Faculty of Medical Science and Research, Nadiad, Gujarat, India, over 12 months from August 1, 2024, to August 31, 2025. The study was approved by the Institutional Ethics Committee (protocol number: NDDFMSR/IEC/12/2024). 

Eligibility criteria

Patients aged 18 years and above with a confirmed diagnosis of acute STEMI as judged by clinical presentation and electrocardiographic criteria under the Fourth Universal Definition of Myocardial Infarction [13] and a hospital admission within 24 hours of onset of the symptoms were included. Patients were excluded if they had known cardiac dysfunction or structural heart disease, chronic systemic illness or disorder affecting vitamin D metabolism, recent administration of vitamin D or drugs affecting vitamin D metabolism, hemodynamic instability, or were unable to undergo echocardiographic assessment. Patients who declined to offer informed consent were also excluded.

Sample size calculation

Sample size estimation was performed using the standard single-proportion method, based on the findings of Avarebeel et al., who reported a 72% prevalence of vitamin D deficiency among patients with acute STEMI [10]:

$n = \frac{Z^2 \times p \times (1 - p)}{d^2}$

The estimated minimum sample size was 121 patients, based on an expected prevalence of 72% (p = 0.72), a 95% confidence level (Z = 1.96), and an absolute precision of 8% (d = 0.08). To improve the statistical robustness of the study and compensate for missing data, incomplete laboratory investigations, or non-evaluable echocardiographic assessments, the sample size was inflated by approximately 20-25%, and a final sample size of 150 patients was targeted for inclusion.

Data collection and clinical evaluation

All data were collected during the index hospitalization. A pre-structured data collection form was used to collect demographic data, cardiovascular risk factors, infarct territory (anterior vs. non-anterior), Killip class at presentation, and mode of reperfusion therapy. At admission, body mass index (BMI) was calculated.

Laboratory investigations and vitamin D categorization

Sampling of venous blood was done within 24 hours after hospitalization and before any vitamin D supplement. Serum was transferred immediately and kept in normal conditions. Vitamin D levels were quantified through a chemiluminescent immunoassay (CLIA) (CL-900i analyzer; Mindray Bio-Medical Electronics Co., Ltd., Shenzhen, Guangdong, China) with the necessary internal and external control groups. Patients were categorized into three groups based on the concentration of vitamin D as follows: (i) Deficient: <20 ng/mL, (ii) Insufficient: 20-29 ng/mL, and (iii) Normal (sufficient): ≥30 ng/mL.

Laboratory parameters, including serum creatinine, glomerular filtration rate, fasting blood glucose, calcium, phosphate, and lipid profile, were analyzed using Erba Biochemical analyzer XL 200 and Erba kits (Erba Lachema s.r.o., Brno, Czech Republic). hs-CRP was evaluated using the cobas® e 411 analyzer (Roche Holding AG, Basel, Switzerland).

Echocardiographic assessment

Transthoracic echocardiography was carried out on all the patients with an Epiq 7C echocardiography system (Koninklijke Philips N.V., Amsterdam, Netherlands) between 48 and 72 hours after they were admitted, when their hemodynamic condition was stable. The echocardiographic measures were done using experienced cardiologists who were not aware of the vitamin D status. The biplane Simpson method was used for LVEF. It was considered to be LV systolic dysfunction if LVEF was less than 50%. Other echocardiographic measures, such as regional WMSI and TAPSE, were measured where possible.

Outcome measures

The main outcome was the presence of LV systolic dysfunction. Secondary outcomes included variation in continuous measures of LVEF and WMSI across vitamin D categories and the incidence of in-hospital complications.

Statistical analysis

Data analysis was done with the help of IBM SPSS Statistics for Windows, version 29.0 (IBM Corp., Armonk, New York, United States). Continuous variables were given in mean and standard deviation (SD), and categorical variables were given in frequencies and percentages. One-way analysis of variance (ANOVA) of continuous variables and chi-square tests or Fisher's exact tests of categorical variables were the methods of between-group comparison. The relationship between vitamin D levels and echocardiographic parameters was assessed using the Pearson correlation coefficient. Independent variables of LV systolic dysfunction were determined through multivariable logistic regression analysis. A p-value of less than 0.05 was significant.

## Results

A total of 150 patients with acute STEMI were included in this study. Table 1 demonstrates vitamin D distribution among the study population. Vitamin D insufficiency was the most prevalent category (46.7%), followed by normal vitamin D status (33.3%), while 20.0% of patients were vitamin D deficient. This distribution indicates a high burden of suboptimal vitamin D levels among STEMI patients.

**Table 1:** Vitamin D status of study participants (N=150)

Vitamin D Category	Frequency (Percentage)
Insufficient (20–29 ng/mL)	70 (46.7)
Normal (≥30 ng/mL)	50 (33.3)
Deficient (<20 ng/mL)	30 (20.0)

Table 2 summarizes the baseline demographic and clinical profile of the study population (n=150) stratified by vitamin D category. The mean age was comparable across groups (p=0.46). The majority of the patients were male (n=112, 74.7%). BMI also did not differ significantly between groups (p=0.83). Diabetes mellitus showed a significant intergroup difference, being more frequent in the deficient group (n=18, 60.0%) compared with the insufficient (n=28, 40.0%) and normal groups (n=20, 40.0%) (p=0.04). Hypertension and dyslipidemia were similarly distributed across vitamin D categories (p=0.79 and p=0.83, respectively). Current smoking differed significantly across groups, with the highest prevalence observed among vitamin D-deficient patients (n=20, 66.7%) compared with insufficient (n=22, 31.4%) and normal (n=18, 36.0%) categories (p=0.01). Anterior wall myocardial infarction (MI) also showed a significant distribution difference, being lowest in the normal group (n=18, 36.0%) and highest in the deficient group (n=25, 83.3%), while the insufficient group had 36 (51.4%) cases (p=0.001).

**Table 2:** Baseline demographic and clinical characteristics by vitamin D category ^a^ one-way ANOVA; ^b^ Chi square (χ²) test; ^*^ significant (p <0.05) BMI: body mass index; MI: myocardial infarction

Variable	Total (n=150)	Insufficient (n=70)	Normal (n=50)	Deficient (n=30)	Test statistic (F / χ²)	p value
Age (years), mean ± SD	56.8 ± 9.7	56.9 ± 9.5	55.7 ± 9.0	58.4 ± 10.6	F = 0.75	0.46^a^
Male sex, n (%)	112 (74.7)	52 (74.3)	37 (74.0)	23 (76.7)	χ² = 0.08	0.96^b^
BMI (kg/m²), mean ± SD	26.4 ± 3.1	26.3 ± 3.0	26.6 ± 3.1	26.2 ± 3.4	F = 0.20	0.83^a^
Diabetes mellitus, n (%)	66 (44.0)	28 (40.0)	20 (40.0)	18 (60.0)	χ² = 3.90	0.04^ b*^
Hypertension, n (%)	74 (49.3)	33 (47.1)	25 (50.0)	16 (53.3)	χ² = 0.47	0.79^ b^
Dyslipidaemia, n (%)	64 (42.7)	30 (42.9)	20 (40.0)	14 (46.7)	χ² = 0.37	0.83^ b^
Current smoker, n (%)	60 (40.0)	22 (31.4)	18 (36.0)	20 (66.7)	χ² = 11.37	0.01^ b*^
Anterior wall MI, n (%)	79 (52.7)	36 (51.4)	18 (36.0)	25 (83.3)	χ² = 16.93	0.001^ b*^

Table 3 compares laboratory parameters across vitamin D categories and demonstrates significant intergroup differences in key biochemical and inflammatory markers. Mean serum 25(OH)D levels showed a clear gradient (p<0.001). Serum calcium was significantly lower in the deficient group (8.12 ± 0.60 mg/dL) compared with insufficient (8.34 ± 0.49) and normal (8.56 ± 0.43) (p=0.003). Inflammatory burden, assessed by hs-CRP, was highest among deficient patients (13.6 ± 5.4 mg/L) versus insufficient (9.12 ± 4.86) and normal (7.68 ± 4.35) (p<0.001). Renal function also differed significantly, with lower estimated glomerular filtration rate (eGFR) in the deficient group (76.51 ± 15.32 mL/minute/1.73 m²) compared with insufficient (82.36 ± 14.0 mL/minute/1.73 m²) and normal (83.45 ± 13.3 mL/minute/1.73 m²) (p=0.04). Phosphate and creatinine did not differ significantly across the vitamin D level categories (p=0.14 and p=0.08, respectively).

**Table 3:** Laboratory profile across vitamin D categories Intergroup comparisons were performed using one-way ANOVA. F value denotes the ANOVA test statistic. A two-sided p <0.05 was considered statistically significant (*) 25(OH)D: 25-hydroxyvitamin D; eGFR: estimated glomerular filtration rate; hs-CRP: high-sensitivity C-reactive protein

Variable (mean±SD)	Total (n=150)	Insufficient (n=70)	Normal (n=50)	Deficient (n=30)	F value	p value
25(OH)D (ng/mL), mean ± SD	27.0 ± 8.7	24.2 ± 2.4	36.1 ± 4.3	14.7 ± 3.1	424.95	<0.001*
Calcium (mg/dL), mean ± SD	8.38 ± 0.52	8.34 ± 0.49	8.56 ± 0.43	8.12 ± 0.60	7.63	0.003*
Phosphate (mg/dL), mean ± SD	3.58 ± 0.63	3.60 ± 0.60	3.65 ± 0.61	3.40 ± 0.72	1.57	0.14
Creatinine (mg/dL), mean ± SD	0.94 ± 0.27	0.92 ± 0.26	0.90 ± 0.23	1.02 ± 0.32	2.11	0.08
eGFR (mL/min/1.73m²), mean ± SD	81.2 ± 14.1	82.6 ± 14.0	83.45 ± 13.3	76.51 ± 15.32	2.44	0.04*
hs-CRP (mg/L), mean ± SD	10.1 ± 5.3	9.1 ± 4.8	7.6 ± 4.1	13.6 ± 5.4	15.70	<0.001*

Table 4 shows significant differences in echocardiographic parameters across vitamin D categories. Mean LVEF was highest in the normal group (54.00 ± 6.00%), followed by the insufficient group (51.60 ± 6.80%), and lowest in the deficient group (44.80 ± 7.90%), with a statistically significant intergroup difference (p < 0.001). LV systolic dysfunction (LVEF <50%) was observed in 12 (24.00%) normal, 24 (34.29%) insufficient, and 30 (100.00%) deficient patients (overall, n=66, 44.00%), showing a significant association with vitamin D status (p < 0.001). WMSI was significantly higher in the deficient group (1.82 ± 0.31) compared with the insufficient (1.53 ± 0.27) and normal (1.45 ± 0.23) groups (p < 0.001), indicating more extensive regional wall motion abnormalities. TAPSE was also lowest in the deficient group (16.20 ± 2.50 mm) compared with the insufficient (18.30 ± 2.10 mm) and normal (19.00 ± 2.20 mm) groups, and this difference was statistically significant (p < 0.001). Overall, worsening vitamin D status was associated with poorer LV systolic performance, greater wall motion abnormality, and reduced RV systolic function.

**Table 4:** Echocardiographic findings across the vitamin D categories Vitamin D categories were defined as Deficient (<20 ng/mL), Insufficient (20–29 ng/mL), and Normal (≥30 ng/mL). ^a^ one-way ANOVA; ^b^ Chi square (χ²) test; *significant (p <0.05). LVEF: left ventricular ejection fraction; LV: left ventricular; WMSI: Wall Motion Score Index; TAPSE: tricuspid annular plane systolic excursion

Variable	Total (n=150)	Insufficient (n=70)	Normal (n=50)	Deficient (n=30)	Test statistic (F / χ²)	p value
LVEF (%), mean ± SD	50.6 ± 7.4	51.6 ± 6.8	54.0 ± 6.0	44.8 ± 7.9	F = 23.84	<0.001^a*^
LV systolic dysfunction (LVEF <50%), n (%)	66 (44.0)	24 (34.3)	12 (24.0)	30 (100.0)	χ² = 62.31	<0.001^b^*
WMSI, mean ± SD	1.59 ± 0.30	1.53 ± 0.27	1.45 ± 0.23	1.82 ± 0.31	F = 26.19	<0.001^a*^
TAPSE (mm), mean ± SD	18.0 ± 2.4	18.3 ± 2.1	19.0 ± 2.2	16.2 ± 2.5	F = 14.78	<0.001a*

Table 5 demonstrates significant correlations between serum 25(OH)D levels and indices of ventricular function and inflammation. Serum vitamin D showed a strong positive correlation with LVEF (r = 0.62; p < 0.001), indicating better LV systolic function with higher vitamin D levels. There was a strong negative correlation between 25(OH)D and WMSI (r = −0.58; p < 0.001), which means that lower vitamin D levels were linked to more severe regional wall motion abnormality. Vitamin D was also positively correlated with TAPSE (r = 0.46; p < 0.001), reflecting improved right ventricular systolic function with higher 25(OH)D levels. Additionally, 25(OH)D demonstrated a significant negative correlation with hs-CRP (r = −0.52; p < 0.001), supporting an association between hypovitaminosis D and increased systemic inflammation. 

**Table 5:** Pearson correlation analysis between serum 25(OH)D levels and echocardiographic/inflammatory parameters in STEMI patients Pearson’s correlation coefficient (r) and corresponding two-tailed p values for associations between serum 25(OH)D and cardiac functional indices. A positive r indicates a direct association, whereas a negative r indicates an inverse association. * Significant (p < 0.05) 25(OH)D: 25-hydroxyvitamin D; LVEF: left ventricular ejection fraction; WMSI: Wall Motion Score Index; TAPSE: tricuspid annular plane systolic excursion; hs-CRP: high-sensitivity C-reactive protein; STEMI: ST-segment elevation myocardial infarction

Correlation Pair	r value	p value
25(OH)D vs LVEF	0.62	<0.001*
25(OH)D vs WMSI	-0.58	<0.001*
25(OH)D vs TAPSE	0.46	<0.001*
25(OH)D vs hs-CRP	-0.52	<0.001*

Table 6 demonstrates the independent variables of LV systolic dysfunction after adjustment for major clinical covariates. Vitamin D deficiency was strongly associated with LV dysfunction (adjusted OR (aOR) 6.20; 95%CI 2.35-16.36; p < 0.001). Diabetes mellitus independently increased the odds of LV dysfunction (aOR 2.85; 95%CI 1.36-5.94; p = 0.005). Anterior wall MI showed a robust association (aOR 4.10; 95%CI 1.88-8.93; p < 0.001). Elevated hs-CRP (>10 mg/L) also remained significant (aOR 3.05; 95%CI 1.45-6.42; p = 0.003), while vitamin D insufficiency did not reach statistical significance (p = 0.14).

**Table 6:** Multivariable logistic regression analysis for the variables associated with LV systolic dysfunction (LVEF <50%) among STEMI patients (N = 150) Results are presented as adjusted odds ratios (aOR) with 95% confidence intervals (CI) and two-sided p values. Vitamin D status was entered as a three-level categorical variable with normal vitamin D (≥30 ng/mL) as the reference category. *significant (p value <0.05) aOR: adjusted odds ratio; CI: confidence interval; LVEF: left ventricular ejection fraction; hs-CRP: high-sensitivity C-reactive protein; MI: myocardial infarction; STEMI: ST-segment elevation myocardial infarction

Variables	aOR	95% CI	p-value
Vitamin D status			
Normal (≥30 ng/mL)	1.00	Reference	Not Applicable
Insufficient (20–29 ng/mL)	1.85	0.82 – 4.17	0.14
Deficient (<20 ng/mL)	6.20	2.35 – 16.36	<0.001*
Diabetes mellitus (Yes vs No)	2.85	1.36 – 5.94	0.005*
Anterior wall MI (Yes vs No)	4.10	1.88 – 8.93	<0.001*
hs-CRP >10 mg/L (Yes vs No)	3.05	1.45 – 6.42	0.003*
Age (per 10-year increase)	1.22	0.93 – 1.60	0.15

## Discussion

LV systolic dysfunction is still a key prognostic factor after acute STEMI, despite the provision of early reperfusion therapy. Current research shows a correlation between lower serum vitamin D levels and compromised LVEF in acute STEMI patients. Vitamin D-deficient patients were characterized by a significantly higher rate of LV systolic dysfunction, higher WMSI, and greater inflammatory markers than patients with normal levels of vitamin D. Moreover, multivariate analysis observed that deficiency of vitamin D showed a significant association with ventricular dysfunction, indicating that hypovitaminosis D helps in the negative myocardial remodeling development in the initial period of infarction.

Vitamin D has progressively been considered a cardiovascular regulatory hormone and not just a bone metabolite mediator. The receptors of vitamin D were observed in cardiomyocytes, vascular smooth muscle cells, and endothelial cells. The activation of these receptors also affects myocardial contractility, calcium transport, and neurohormonal activity. It has been demonstrated through experimental studies that vitamin D stops the renin-angiotensin-aldosterone system, oxidative stress, and regulates inflammatory cytokines, which are significant factors of myocardial injury and post-infarction remodeling [14-16]. 

Clinical studies observed that vitamin D deficiency is more prevalent in patients with acute coronary syndromes. Baktir et al. also stated that lower serum 25(OH)D levels were significantly related to the severity of CAD, as patients with acute coronary syndrome had higher SYNTAX (SYNergy between percutaneous coronary intervention with TAXus and cardiac surgery) scores, indicating more extensive myocardial involvement and a poorer prognosis [17]. On the same note, Kheiri et al. found that deficiency of vitamin D is independently correlated with a risk of cardiovascular events and mortality due to its role in impairing endothelial dysfunction, inflammation, and neurohormonal activation [18]. These observations are in line with our results because almost two-thirds of the population under study had inadequate or lacking vitamin D levels, which indicates that hypovitaminosis D is very common among patients presenting with STEMI.

Vitamin D was examined in observational and mechanistic studies with respect to its relationship with ventricular systolic function. Vitamin D deficiency is linked to reduced remodeling of the cardiovascular system, poor myocardial contractility, and heightened inflammatory response, all of which can be the cause of ventricular dysfunction following ischemic stroke. Recently, Haider et al. conducted a review that showed vitamin D deficiency correlates with increased cardiovascular disease risk and myocardial dysfunction due to the impact on the inflammatory processes, endothelial dysfunction, and neurohormonal activation [19]. Fallo et al. reported a relationship between the concentration of serum 25(OH)D and LV hypertrophy [20]. Similarly, Nardin et al. explained how vitamin D regulates the remodeling of the myocardium, endothelial dysfunction, and thrombosis, which are major mechanisms involved in impairment of the ventricles following an infarction [21]. Khademvatani et al. have reported that vitamin D deficiency is an independent predictor of increased odds of ventricular dysfunction and increased inflammatory markers in acute coronary syndrome patients [22]. These reports are in line with our results, which show that serum vitamin D levels positively correlate significantly with LVEF and negatively with WMSI and hs-CRP, which supports the hypothesis that inflammation-mediated myocardial injury could be one of the underlying reasons hypovitaminosis D is associated with reduced systolic function.

Inflammation contributes to the key role in myocardial infarction and ventricular remodeling post-STEMI, and elevated levels of CRP have been associated with excessively large areas of the infarct, worse ventricular performance, and mortality. Vitamin D has endothelium receptors, vascular smooth muscle receptors, and cardiomyocyte receptors [23]. Vitamin D also controls immunity when it inhibits nuclear factor-kB signaling and pro-inflammatory cytokines [24]. Higher levels of hs-CRP in patients with vitamin D deficiency in this study were in support of hypovitaminosis D in the stimulation of post-infarction inflammation.

In the multivariable model, vitamin D deficiency remained strongly associated with LV systolic dysfunction even after adjustment for key clinical covariates (diabetes mellitus, anterior wall MI, inflammatory status as hs-CRP, and age). However, because all vitamin D-deficient patients had LV dysfunction, the dataset demonstrates potential complete separation, which can inflate effect estimates and reduce stability in conventional logistic regression. Therefore, the aORs should be interpreted as reflecting a strong association rather than a precise causal effect size. Importantly, the direction of the multivariable findings is supported by consistent evidence from unadjusted comparisons and correlation analysis, where lower 25(OH)D levels were associated with lower LVEF and higher WMSI and hs-CRP. Future studies should validate these findings in larger cohorts and apply separation-robust approaches (e.g., Firth penalized regression) and infarct-severity adjustment (Killip class, ischemic time, reperfusion variables, and peak biomarker). Past investigations showed low levels of vitamin D are connected with risk of cardiovascular and myocardial infarction and unfavorable ventricular remodeling following infarction, as well as its participation in post-MI ventricular dysfunction [25-27].

From a clinical standpoint, serum 25(OH)D measurement is inexpensive and widely available, and identification of deficiency during STEMI admission may assist risk stratification when interpreted alongside established variables such as infarct territory and inflammatory burden. Nonetheless, therapeutic implications should be framed cautiously. Mirhosseini et al. carried out a systematic review and meta-analysis study, demonstrating that Vitamin D supplements can lower cardiovascular risk factors [28].

The study has several limitations. First, the cross-sectional design precludes inference of causality or temporality, and reverse causation due to the acute inflammatory response; lowering circulating vitamin D cannot be excluded. Second, LV dysfunction occurred in all vitamin D-deficient patients, raising the possibility of complete separation, which may lead to unstable or inflated regression estimates in standard logistic regression. Third, residual confounding related to infarct severity (e.g., Killip class, time-to-reperfusion, peak troponin/creatine kinase-myocardial band (CK-MB) may persist despite adjustment for major clinical variables. Fourth, this was a single-center tertiary care study, which may limit generalizability to other settings and populations. Fifth, very severe presentations (e.g., early deaths or patients too unstable to undergo echocardiography) may be under-represented, introducing potential selection/survivorship bias. Sixth, key determinants of vitamin D status, such as seasonality, sunlight exposure, physical activity, dietary intake, and prior supplementation, were not measured and could contribute to residual confounding. Seventh, multiple comparisons were performed across several outcomes and correlations, and in the absence of formal multiplicity correction, secondary p-values should be interpreted cautiously.

## Conclusions

Vitamin D deficiency was strongly associated with early left ventricular systolic dysfunction and adverse echocardiographic indices among patients hospitalized with acute STEMI. The observed relationships were consistent across group comparisons and correlation analysis and were biologically plausible in the context of inflammation-mediated myocardial injury. Although multivariable modelling suggested an independent association, the effect estimates should be interpreted cautiously due to potential residual confounding and possible separation-related instability in the deficient subgroup. Overall, serum 25(OH)D may serve as a clinically accessible risk marker to support early risk stratification in STEMI when interpreted alongside established severity indicators. Prospective cohort studies with standardized infarct-severity adjustment and follow-up assessment of ventricular function, and randomized trials evaluating correction of vitamin D deficiency in STEMI, are warranted to clarify prognostic value and therapeutic implications.
